# An unwelcome inheritance: childhood obesity after diabetes in pregnancy

**DOI:** 10.1007/s00125-023-05965-w

**Published:** 2023-07-13

**Authors:** Claire L. Meek

**Affiliations:** 1 Wellcome Trust MRC Institute of Metabolic Science, University of Cambridge, Cambridge, UK; 2 Cambridge University Hospitals NHS Foundation Trust, Cambridge, UK

**Keywords:** Childhood obesity, Gestational diabetes, Metabolism, Pregnancy, Review

## Abstract

Diabetes in pregnancy affects 20 million women per year and is associated with increased risk of obesity in offspring, leading to insulin resistance and cardiometabolic disease. Despite the substantial public health ramifications, relatively little is known about the pathophysiological mechanisms underlying obesity in these high-risk children, which creates a barrier to successful intervention. While maternal glucose itself is undeniably a major stimulus upon intrauterine growth, the degree of offspring hyperinsulinism and disturbed lipid metabolism in mothers and offspring are also likely to be implicated in the disease process. The aim of this review is to summarise current understanding of the pathophysiology of childhood obesity after intrauterine exposure to maternal hyperglycaemia and to highlight possible opportunities for intervention. I present here a new unified hypothesis for the pathophysiology of childhood obesity in infants born to mothers with diabetes, which involves self-perpetuating twin cycles of pancreatic beta cell hyperfunction and altered lipid metabolism, both acutely and chronically upregulated by intrauterine exposure to maternal hyperglycaemia.

**Graphical Abstract:**

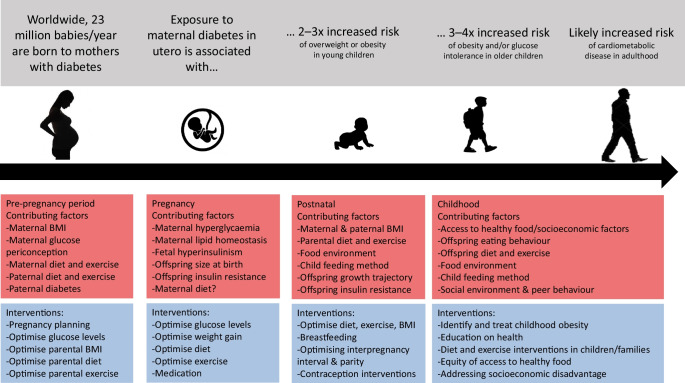

**Supplementary Information:**

The online version of this article 10.1007/s00125-023-05965-w contains a slide of the figure for download, which is available to authorised users.


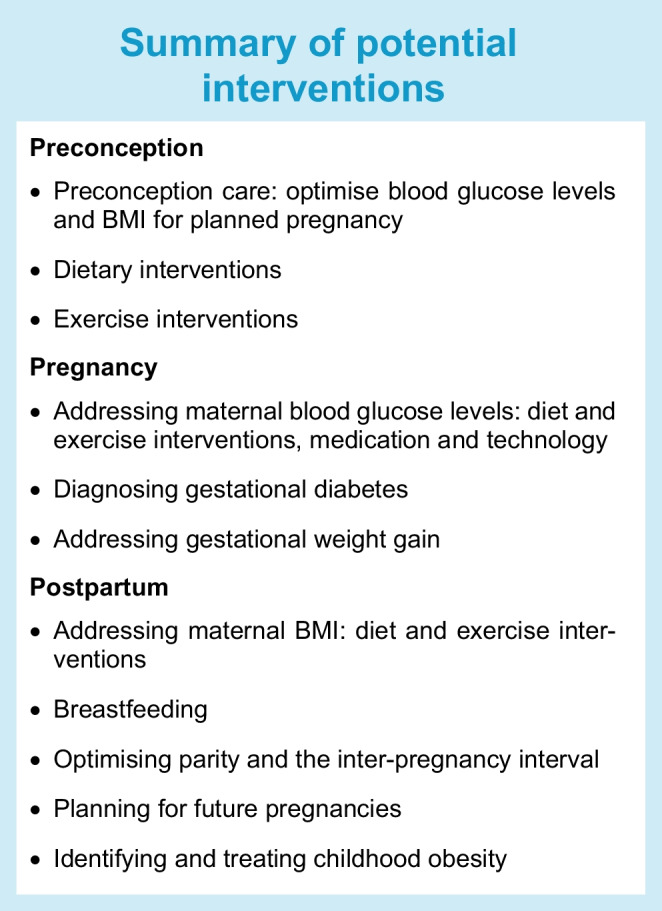


## Introduction

Diabetes in pregnancy affects one in six pregnancies internationally and is associated with short-term and long-term health sequelae for both mother and child [1, 2]. Infants born to mothers with diabetes are commonly large-for-gestational-age at birth and have a higher risk of obesity in childhood [2, 3]. Obesity in childhood has many deleterious consequences upon future cardiometabolic health, including early-onset insulin resistance [4] and type 2 diabetes [5]. Rapid childhood growth is also associated with increased risk of type 1 diabetes [6]. The early development of obesity in children with existing environmental and genetic susceptibilities to diabetes should be a major public health concern [7].

Unfortunately, very few interventions with proven effectiveness are available to reduce the risk of obesity in high-risk children [5]. Barriers to successful intervention include knowledge gaps about the mechanisms of disease, the optimal timing for effective action and the most suitable short-term or long-term measures to modify the disease process. The aim of this review is to summarise current understanding of the pathophysiology of childhood obesity after intrauterine exposure to maternal hyperglycaemia and to highlight possible opportunities for intervention.

## Childhood obesity after diabetes in pregnancy: the scale of the problem

There is a growing consensus that the risk of obesity (BMI ≥95th centile for age [5]) is elevated in offspring exposed to gestational diabetes, type 1 diabetes or type 2 diabetes in utero [8, 9], although the data remain controversial [10, 11]. Many studies are limited by incomplete adjustment for important confounding factors such as maternal obesity or socioeconomic status, insufficient sample size or inadequate duration of follow-up. Maternal obesity is a particularly important confounding factor and is likely to exert effects both genetically and environmentally upon intrauterine development and postnatal lifestyle and behaviour. A recent metanalysis identified that maternal obesity was associated with offspring overweight or obesity in early-, mid- and late childhood with odds ratios (OR) of 2.43, 3.12 and 4.47, respectively [12].

Despite these limitations, there is consistent evidence of an association between maternal diabetes and child obesity, described in diverse populations including from China [13], Denmark [14], Sweden [15], Turkey [16], South Korea [17] and in the multi-ethnic Hyperglycemia and Adverse Pregnancy Outcomes (HAPO) cohort [10]. There is also emerging evidence of a dose-dependent effect of maternal hyperglycaemia upon offspring obesity, with risk increasing proportionately with maternal glucose concentrations in pre-gestational and gestational diabetes [11, 14]. This dose-dependent effect is also evident in pregnancies with lower levels of hyperglycaemia in pregnancy, below the diagnostic thresholds for gestational diabetes [18, 19], with elevated obesity rates in affected offspring, even after adjustment for maternal BMI. A sibling study suggests that the effect of maternal hyperglycaemia upon offspring obesity risk is likely to be primarily a developmental effect [15].

## Childhood obesity after diabetes in pregnancy: body composition

The importance of developmental effects upon obesity risk suggests that large-for-gestational-age and childhood obesity may be two manifestations of the same mechanistic process. Size at birth is an important determinant of adiposity in adults [20, 21], and large-for-gestational-age is associated with a twofold increase in the rate of obesity in childhood [9, 22]. Excessive growth postnatally (not unique to diabetes in pregnancy) also contributes to child obesity [23].

Although increases in child BMI are evident, it is unclear how this relates to body composition. BMI has limitations as a marker of adiposity in children, especially during active growth. The risk of childhood adiposity after gestational diabetes was assessed in over 4000 children aged 10 to 14 years old from the HAPO cohort [2, 24, 25]. Associations were identified between gestational diabetes and child obesity, percentage body fat and sum of skinfold thickness, independent of maternal BMI [2]. These data suggest that the increase in child BMI represents a true change in body composition, characterised by increased fat mass.

Questions still remain about fat distribution and type (white, brown and beige adipose cells) after exposure to intrauterine hyperglycaemia. Data from the multi-ethnic Growing Up in Singapore Towards healthy Outcomes (GUSTO) cohort suggest that the increased abdominal circumference seen in hyperglycaemia-exposed neonates is due to increased deep and subcutaneous adipose tissue, with increases in liver fat [26]. Santos and colleagues found no association between maternal diabetes and infant fat distribution using anthropometry [27] (Generation XXI, Portugal; *n*=4747). However, rapid fetal growth, a key characteristic of intrauterine exposure to maternal diabetes [28], has been associated with specific increases in central fat accretion [29] and linked to future risk of liver steatosis [30].

## Childhood obesity after diabetes in pregnancy: does the type of diabetes matter?

Although type 1, type 2 and gestational diabetes have distinct mechanistic causes, they all demonstrate similar associations with childhood obesity, broadly proportionate to the severity of hyperglycaemia in pregnancy [11, 14, 18, 19]. This suggests that there is a common pathway to childhood obesity, regardless of diabetes aetiology. Offspring of mothers with type 2 diabetes in pregnancy or gestational diabetes may be exposed to less severe hyperglycaemia but may have additional genetic and socioeconomic factors which predispose to obesity.

## Part 1: potential mechanisms

### Maternal glucose homeostasis and offspring body size and composition

Maternal glucose is the main fuel substrate for both fetus and placenta during a healthy pregnancy [31], with a direct effect upon offspring intrauterine growth in gestational diabetes, type 1 diabetes and type 2 diabetes [32]. We previously demonstrated strong associations between birthweight and maternal hyperglycaemia, assessed using continuous glucose monitoring metrics or biochemical measures [33].

The Pedersen hypothesis from 1952 states that a fetus will develop hyperinsulinism in response to maternal hyperglycaemia, as maternal glucose can travel freely across the placenta, while maternal insulin cannot [34]. Abundant glucose from the maternal circulation and abundant insulin from the fetal pancreas produce an environment with enhanced glycolysis and plentiful cellular energy for fetal growth.

### Maternal lipid homeostasis and offspring body size and composition

While established data highlight the importance of maternal glucose, not maternal lipids, in the development of offspring adipose tissue [35], there is emerging evidence that maternal hyperglycaemia may also directly or indirectly increase offspring adiposity through altered lipid metabolism. Using the UK Pregnancies Better Eating and Activity Trial (UPBEAT) cohort, we recently identified that lipid species associated with de novo lipogenesis (species containing fatty acids 16:0, 16:1, 18:0 and 18:1) were elevated in mothers with gestational diabetes, and directly associated with offspring adiposity (abdominal circumference) independently of maternal hyperglycaemia [36]. These findings were further supported by evidence in women with type 1 diabetes, which demonstrated strong associations between offspring skinfold sum and lipid species in maternal serum, independently of maternal glucose [37]. The strongest independent associations were identified with species including fatty acids 16:0 or 18:1, consistent with increased or upregulated de novo lipogenesis. A mediation analysis suggested that maternal lipids are important but do not solely mediate the relationship between maternal hyperglycaemia and offspring adiposity [37].

Recent work has identified a reduction in brown adipose tissue (BAT) in mothers with gestational diabetes, with reduction in concentrations of BAT-derived adipokines, neuregulin-4 [38, 39] and angiopoietin-like protein 8 (ANGPTL8) [40]. Although these data merit further investigation, the significance of maternal or offspring BAT upon offspring obesity risk in childhood remains unclear.

### The fetal response to maternal hyperglycaemia: offspring insulin secretion

Hyperinsulinism, or augmented fetal beta cell function, is undoubtedly a key pathophysiological mechanism in diabetes in pregnancy and directly associated with many clinical sequelae, including neonatal hypoglycaemia and large-for-gestational age [34, 41]. However, it is difficult to study potential genetic and environmental variations in the fetal response to maternal hyperglycaemia because a meaningful assessment of fetal metabolism in humans is so challenging.

Although it is logical to assume that neonatal hyperinsulinism, neonatal hypoglycaemia and large-for-gestational age are closely related sequelae of late pregnancy hyperglycaemia, our recent metabolomics analysis of the Continuous Glucose Monitoring in Women with Type 1 Diabetes in Pregnancy Trial (CONCEPTT) cohort suggests there are important differences in these conditions and in the timing of onset [37]. For example, neonatal hypoglycaemia was associated with marked increases in lipid abundance in maternal blood in the first trimester, suggesting maternal lipolysis (e.g. due to insufficient insulin dosing or energy restriction due to nausea and vomiting in pregnancy). Neonatal hyperinsulinism was also associated with first-trimester changes in maternal metabolites, showing positive associations with phenolic compounds (saccharin, metabolites from phenolic compounds in tea, coffee, chocolate and olives). Taken together, these findings suggest that maternal metabolism and nutrition at the time of fetal pancreatic development may have an important effect upon offspring pancreatic function, but these data require corroboration with other cohorts.

New translational opportunities may arise from a better understanding of the determinants of fetal hyperinsulinism longitudinally during pregnancy, especially if fetal metabolism can be measured and monitored. Recent work in the CONCEPTT cohort identified that pregnancies with the most hyperinsulinaemic offspring had a third-trimester increase in C-peptide in maternal blood, unexpected in women with no evidence of beta cell function at baseline, providing an opportunity to assess C-peptide as a potential biomarker for fetal hyperinsulinism [42], or fragments of C-peptide, insulin or proinsulin [43].

The longer-term significance of neonatal hyperinsulinism upon children’s metabolic health is also unclear, as current neonatal glucose testing stops 24 h after birth. As clinically relevant episodes of hypoglycaemia seem to happen rarely after the first week of life, the increased insulin production either normalises or becomes less clinically evident, through insulin resistance. Counter-regulatory hormones such as cortisol and glucagon are important in the acute response to neonatal hypoglycaemia and may have a role in determining longer-term insulin sensitivity in childhood.

### The fetal response to maternal hyperglycaemia: offspring insulin resistance

Several studies have identified associations between maternal diabetes and offspring insulin resistance in childhood and adolescence. Boney and colleagues studied children aged 11 years old and identified that exposure to maternal gestational diabetes was associated with insulin resistance (OR 10.4; 95% CI 1.5, 74.4) [44]. The presence of large-for-gestational-age at birth appeared to have an additive effect upon risk [44]. Similar findings were obtained by Sauder et al, who identified increased insulin resistance (18% increase in HOMA-IR) and increased beta cell function (9% increase in HOMA-B) in 10–16 year old children after exposure to intrauterine hyperglycaemia in the Exploring Perinatal Outcomes among Children (EPOCH) study [4]. This study showed that the relationship between maternal diabetes and offspring insulin resistance was not mediated by offspring BMI. The HAPO follow-up study has corroborated these findings [25, 45]. Maternal glucose concentrations in pregnancy showed inverse linear associations between child insulin sensitivity and maternal pregnancy glucose concentrations in the fasting state and 1 h or 2 h after a glucose load. Importantly, these associations were independent of maternal and child BMI [25, 45].

However, since most studies have included older children and adolescents, it remains unclear if insulin resistance is an early or late feature in the development of obesity in children exposed to intrauterine hyperglycaemia. The optimal method for assessing insulin sensitivity is the hyperinsulinaemic–euglycaemic clamp (reference standard) [46]. Surrogate measures such as the hyperglycaemic clamp, the minimal model of the frequently sampled intravenous glucose tolerance test, oral glucose tolerance test or biomarker combinations are more convenient but still unachievable at scale in infants or very young children [46, 47]. Biomarkers associated with insulin resistance have been identified at birth after exposure to gestational diabetes [48] or after antenatal steroids in individuals with type 1 diabetes in pregnancy [49].

### The fetal response to maternal hyperglycaemia: epigenetic influences

Epigenetic changes such as methylation of cytosines in CG dinucleotides (CpG methylation), histone modifications and non-coding RNA may contribute to the effect of the intrauterine environment upon offspring obesity. The Environmental Versus Genetic and Epigenetic Influences on Growth, Metabolism and Cognitive Function in Offspring of Mothers With Type 1 Diabetes (EPICOM) study identified methylation patterns in adolescents exposed to maternal diabetes [50]. Kelstrup and colleagues identified that offspring exposed to gestational diabetes had lower expression of the peroxisome proliferator-activated receptor-γ coactivator-1α (PPARGC1A) in skeletal muscle [51]. Non-coding RNAs such as miRNA and long non-coding RNA (lncRNA) may mediate the effect of maternal diabetes on offspring obesity and pancreatic beta cell dysfunction (reviewed in Saeedi Borujeni et al and Fernandez-Twinn et al [52, 53]). Further progress is this field is limited by technical issues restricting validation across cohorts and the need to adjust for confounders such as maternal obesity [54, 55] and offspring age [56].

### The fetal response to paternal hyperglycaemia: social, genetic and epigenetic influences

Fathers with diabetes pass on genetic and epigenetic traits which influence the metabolic health of their offspring, but they also contribute to social cues for diet and health. Sperm quality, sperm motility, DNA integrity, semen composition and the efficacy of the acrosome reaction are all affected by diabetes [57], but many of the exact mechanisms are unclear in humans. Our own previous work identified that the lipid composition of sperm, required for energy and cell membranes, is associated with sperm motility, suggesting that men’s metabolic health is intrinsically important to fertility [58]. Data from animal models suggest that paternal high-fat diet is associated with changes in DNA methylation and miRNA activity in sperm [59], while exercise may have a beneficial effect [60].

### A unified hypothesis on the effect of intrauterine exposure to hyperglycaemia upon offspring body composition and metabolism

Although we know that maternal and fetal glucose and lipid homeostasis are mechanistically involved in the regulation of body composition after diabetes in pregnancy, many knowledge gaps have yet to be addressed. For example, how is it possible that exposure to maternal hyperglycaemia for a brief period in utero (e.g. 3 months in gestational diabetes, after the period of organogenesis) can have adverse consequences on health across the life course? In addition, why does neonatal metabolism not return to normal once the stimulus of exposure to maternal hyperglycaemia is removed at birth?

While the first trimester is considered the key window for organogenesis in general, the formation and distribution of fetal adipose tissue, a metabolically active endocrine organ, is not confined to the first trimester (reviewed in Desoye and Herrera [35]). Early fat lobules are identifiable in the human fetus from 14 weeks’ gestation, which gradually increase in size due to increasing triglyceride storage throughout the remainder of gestation [35]. Lipid metabolism undoubtedly occurs throughout pregnancy and is likely to be highly regulated, but there is little information on the exact timing and regulation of key processes such as lipid mobilisation or lipid accretion. The third trimester is a key time for lipid deposition in fetal adipose tissue, as demonstrated by the marked differences in body composition at birth and postnatally in preterm infants [61]. With rising maternal insulin resistance through the second trimester, exposure to maternal hyperglycaemia and fetal hyperinsulinaemia in late pregnancy may contribute to increased de novo lipogenesis (making fatty acids) and adipogenesis (making adipose tissue) in offspring. Increased lipoprotein lipase activity in fetal adipose tissue is a potential mechanism behind the enhanced lipid storage [35].

**Fig. 1:**
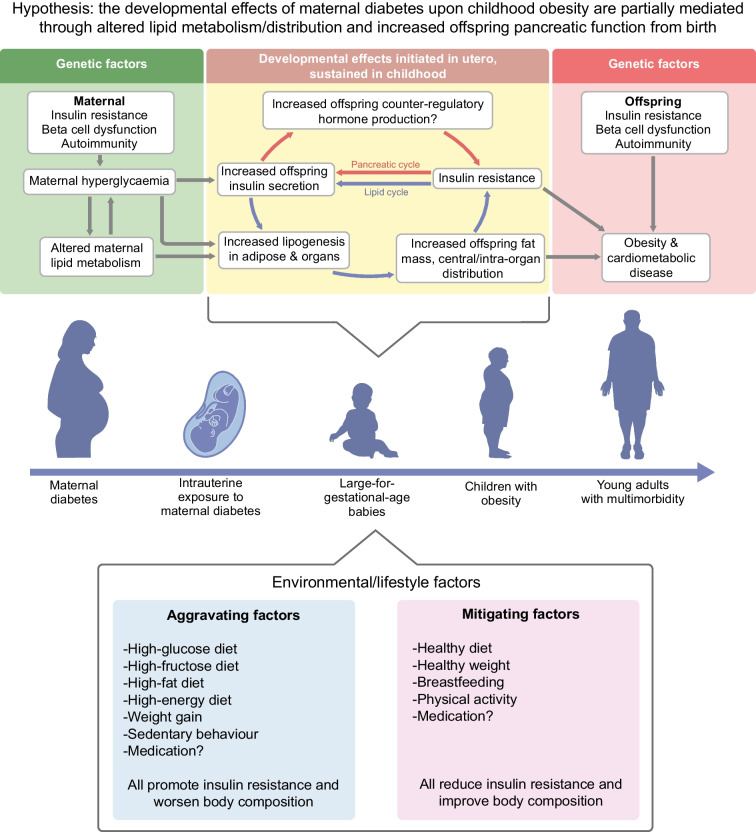
A unifying hypothesis for the development of large-for-gestational-age and childhood obesity after exposure to intrauterine hyperglycaemia. Hypothesis: the developmental effects of maternal hyperglycaemia upon childhood obesity are mediated directly or indirectly through altered offspring lipid metabolism/distribution and increased offspring pancreatic function from birth. These factors exert short-term effects upon body composition but also chronically upregulate key pathways in postnatal life (the lipid cycle and pancreatic cycle), resulting in obesity and cardiometabolic disease. This figure is available as a downloadable slide

One possible explanation for the persisting effect of pregnancy exposure to maternal diabetes is that, in addition to these short-term acute effects, it initiates subtle, chronic changes in body composition predisposing to obesity in childhood. Mechanistically, this could involve two self-perpetuating cycles affecting lipid metabolism and pancreatic function (Fig. 1). Intrauterine exposure to maternal diabetes may result in acute changes in body composition but may also cause subtle upregulation of biological pathways in childhood which support continued lipogenesis and adipogenesis, fed by ongoing excess insulin secretion and resistance, placing children on a trajectory towards later-life cardiometabolic disease.

## Part 2: Potential interventions

Babies born to mothers with diabetes often have multiple risk factors for childhood obesity, which appear to have an additive effect upon risk. Effective interventions targeting maternal hyperglycaemia, maternal obesity and offspring health in the preconception, pregnancy and postnatal periods are needed to prevent childhood cardiometabolic disease. Interventions in the preconception, pregnancy and postnatal periods will be considered in turn.

### Preconception interventions

Pre-pregnancy BMI is a strong modifiable predictor of both birthweight and future childhood obesity [12]. Since many pregnancies are unplanned, and most women do not have access to individualised health promotion advice, preconception interventions are challenging. Population wide strategies [62] and individual diet and lifestyle interventions are reviewed in detail elsewhere [63, 64].

However, preconception advice is more accessible to women with type 1 or type 2 diabetes in pregnancy, but this focuses on improving blood glucose levels and reducing risks of congenital anomalies, rather than reducing rates of childhood obesity [32, 65]. A UK-wide national audit recently found that only 22% of women with type 2 diabetes and 44% of women with type 1 diabetes were taking folic acid preconception (marker of optimal pre-pregnancy care), suggesting that access to preconception advice is still limited [32].

### Pregnancy interventions: addressing hyperglycaemia

Addressing maternal hyperglycaemia through diabetes treatment or prevention may be key to reducing the burden of childhood obesity. Preventing maternal diabetes would be the optimal approach, but there is currently no clear way to prevent gestational diabetes, as trials of lifestyle interventions in pregnancy have had variable results [66, 67]. Effective treatment of gestational diabetes is a helpful contribution to reducing child obesity [19]. The Programming of Enhanced Adiposity Risk in Childhood–Early Screening (PEACHES) study identified that offspring obesity risk was lower in women treated for gestational diabetes, compared with untreated women with hyperglycaemia [19].

Dietary management of gestational diabetes reduces hyperglycaemia in pregnancy and has been associated with lower birthweight, suggesting benefits upon future offspring body composition [68]. A metanalysis demonstrated that women who adhered to one of several dietary approaches to gestational diabetes management had babies 170 g lighter than those who did not follow a specific diet [68]. However, the optimal pregnancy diet to promote good pregnancy outcomes and favourable cardiometabolic health in mothers and offspring is unclear. It is also unknown if dietary strategies for gestational diabetes, type 1 diabetes and type 2 diabetes should be the same. Our RCT, the dietary intervention in gestational diabetes (DiGest) should soon provide some new evidence about the role of maternal energy restriction in short-term and longer-term maternal and offspring outcomes [69].

The role of medication in pregnancy as a means of addressing future child obesity is controversial. Metformin is the most commonly used medication for diabetes in pregnancy, and is economical, convenient and safe. However, a previous meta-analysis identified associations between metformin and accelerated growth postnatally, leading to an increased risk of obesity in childhood, compared with children of women treated with insulin during pregnancy [70]. However, the latest findings on the role of metformin in childhood obesity, from the Metformin in Women with Type 2 diabetes in Pregnancy trial (MITy) follow-up study, have demonstrated no difference in BMI at 2 years of age in children of women who were randomly assigned to receive metformin or placebo during pregnancies affected by type 2 diabetes [71]. While these findings are reassuring, it also suggests that despite treating hyperglycaemia, metformin treatment does not confer any specific benefit upon childhood obesity rates after diabetes in pregnancy. Further follow-up is required to assess the effects of metformin exposure upon BMI in older children.

Novel technologies such as continuous glucose monitoring or closed loop systems have reduced maternal glucose or reduced hypoglycaemic episodes in type 1 diabetes [72, 73] (reviewed in detail [74]). However, technological options are underexplored in type 2 diabetes or gestational diabetes but may improve glycaemic control in the future.

### Pregnancy interventions: addressing maternal gestational weight gain

Some weight gain in pregnancy is expected, but excessive gestational weight gain is very common and has repercussions for women’s BMI for 15 years or more after the pregnancy [75]. Landon and colleagues found that gestational weight gain was strongly related to obesity in children aged 5–10 years old [76], with results confirmed by a meta-analysis [77]. Relatively few studies have attempted weight loss in pregnancy, although several observational studies have identified benefits from restricted gestational weight gain [78]. The DiGest study will provide new data in this field soon [69].

### Postpartum interventions: addressing maternal obesity

Women with excessive gestational weight gain are more likely to retain weight postpartum, which has a long-term effect on maternal BMI [75]. The importance of postpartum weight loss to improve BMI and reduce risk of type 2 diabetes is an area needing much greater attention. Relatively few interventions have shown efficacy in the postpartum period, but lactation remains an important opportunity to improve glucose tolerance, and possibly BMI, in women postnatally. Gunderson and colleagues identified that breastfeeding intensity and duration in women after gestational diabetes were associated with a lower weight gain trajectory in infants [79] and reduced de novo lipogenesis activity in mothers and offspring, downregulating a crucial step in the disease process (Fig. 1). Recent work by Ma and colleagues, in 12 countries internationally, identified that longer breastfeeding duration was associated with reduced risk of obesity in children aged 9–11 years [80].

Internationally, high parity and short inter-pregnancy intervals (<12 months) are substantial contributors to obesity in women. One study in the USA demonstrated an increased risk of obesity in 3422 multiparous women with short inter-pregnancy intervals [81]. Another study has identified a significant effect of parity upon obesity, due in part to cumulative effects of excessive gestational weight gain [82]. Although there is limited evidence on effective interventions, education of women and their partners about reproductive health with improved contraceptive availability may be useful [83].

### Postpartum interventions: addressing child obesity

Guidelines from the Endocrine Society, endorsed by the European Society of Endocrinology and the Paediatric Endocrine Society recognise the lack of good long-term evidence for prevention and amelioration of child obesity [5]. Promoting a healthy diet [84], regular physical activity [85] and a built environment to support a healthy lifestyle [86] are all important, but large, well-controlled studies of specific interventions with prolonged follow-up are lacking [86, 87]. New pharmacological treatments such as semaglutide [88] and an increasing acceptance of bariatric surgery [89, 90] may help adolescents with obesity. The psychological effects of child obesity are significant and also need to be consistently assessed and addressed [5].

## Conclusions

The early development of obesity in children with existing environmental and genetic susceptibilities to type 2 diabetes needs to be addressed to prevent multimorbidity in future generations. Exposure to maternal diabetes and/or obesity in utero is likely to influence offspring body composition, insulin sensitivity and beta cell function. While the mechanisms behind this are underexplored, the complex interplay between maternal and offspring insulin and lipid metabolism are likely to be involved. Effective intervention will require a new focus on maternal health before, during and after pregnancy to halt the intergenerational cycle of obesity.

### Supplementary Information

Below is the link to the electronic supplementary material.

Supplementary file1 (PPTX 178 KB)

## Abbreviations

BAT: Brown adipose tissue
CONCEPTT: Continuous Glucose Monitoring in Women with Type 1 Diabetes in Pregnancy Trial
HAPO: Hyperglycemia and Adverse Pregnancy Outcomes
