# Supplementary material for: An unwelcome inheritance: childhood obesity after diabetes in pregnancy
Source: Diabetologia. 2023 Jul 13;66(11):1961–70. doi: 10.1007/s00125-023-05965-w (PMC10541526; doi:10.1007/s00125-023-05965-w)
Supplement: Supplementary file 1 — Supplementary file1 (PPTX 178 KB) [file 125_2023_5965_MOESM1_ESM.pptx]

## Slide 1
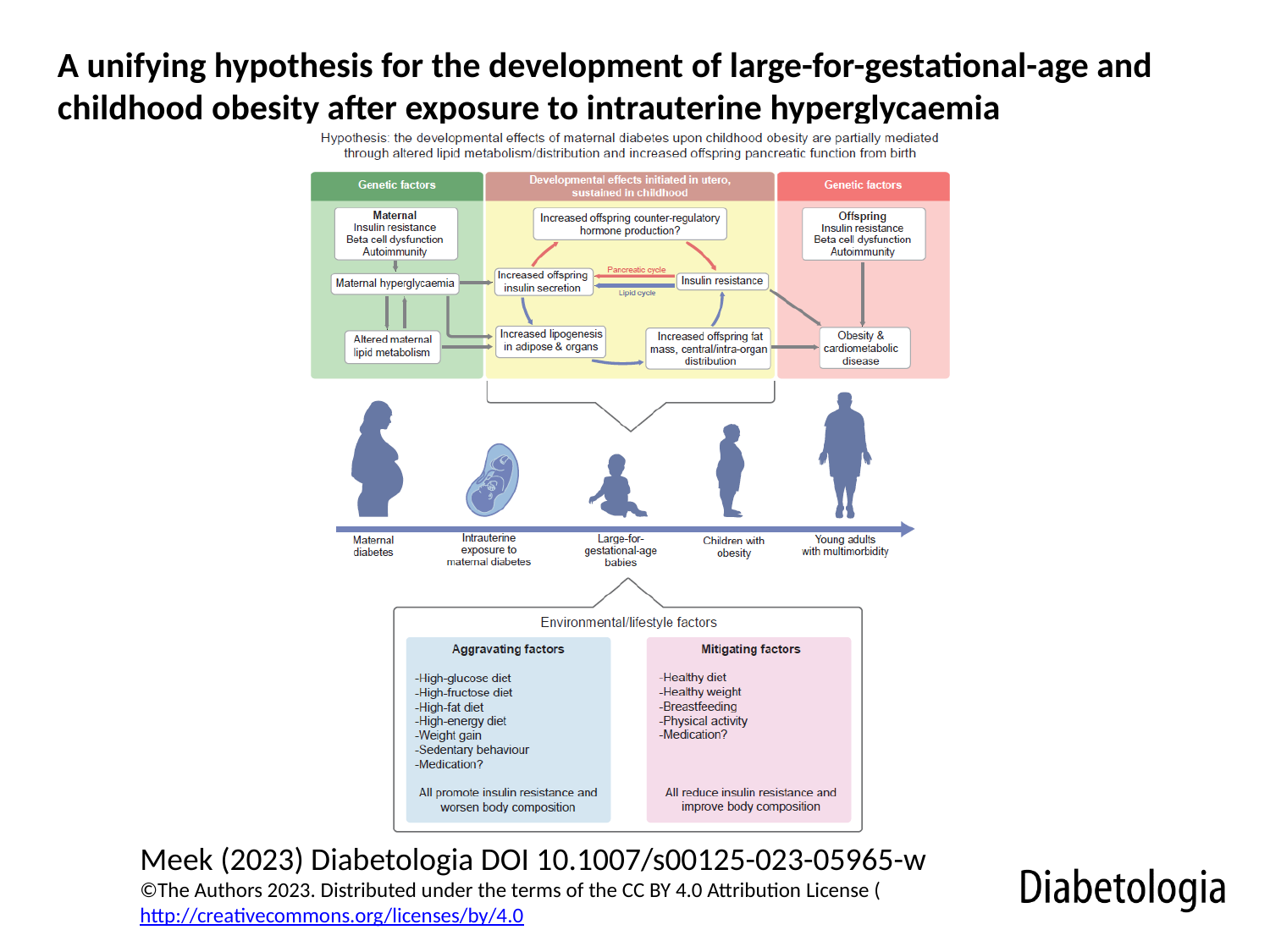

A unifying hypothesis for the development of large-for-gestational-age and childhood obesity after exposure to intrauterine hyperglycaemia
Meek (2023) Diabetologia DOI 10.1007/s00125-023-05965-w
©The Authors 2023. Distributed under the terms of the CC BY 4.0 Attribution License (http://creativecommons.org/licenses/by/4.0
